# Following hip fracture, hospital organizational factors associated with prescription of anti-osteoporosis medication on discharge, to address imminent refracture risk: a record-linkage study

**DOI:** 10.1093/jbmr/zjae100

**Published:** 2024-07-11

**Authors:** Rita Patel, Andrew Judge, Antony Johansen, Muhammad K Javaid, Xavier L Griffin, Tim Chesser, Jill Griffin, Elsa M R Marques, REDUCE Study Group, Celia L Gregson, Antony Johansen, Tim Chesser, Muhammad K Javaid, Xavier L Griffin, Jill Griffin, Elsa M R Marques, Yoav Ben-Shlomo, Sarah Drew, Andrew Judge, Rita Patel, Katie Whale, Yoav Ben-Shlomo, Celia L Gregson

**Affiliations:** Musculoskeletal Research Unit, Translational Health Sciences, Bristol Medical School, University of Bristol, Bristol, BS10 5NB, United Kingdom; Musculoskeletal Research Unit, Translational Health Sciences, Bristol Medical School, University of Bristol, Bristol, BS10 5NB, United Kingdom; Nuffield Department of Orthopaedics, Rheumatology and Musculoskeletal Sciences, University of Oxford, Oxford, OX3 7HE, United Kingdom; NIHR Biomedical Research Centre at University Hospitals Bristol and Weston NHS Foundation Trust and the University of Bristol, BS8 2BN, United Kingdom; Division of Population Medicine, School of Medicine, Cardiff University and University Hospital of Wales, Cardiff, CF14 4XN, United Kingdom; National Hip Fracture Database, Royal College of Physicians, London, NW1 4LE, United Kingdom; Nuffield Department of Orthopaedics, Rheumatology and Musculoskeletal Sciences, University of Oxford, Oxford, OX3 7HE, United Kingdom; Barts Bone and Joint Health, Barts and The London School of Medicine and Dentistry, Queen Mary University of London, London, E1 2AT, United Kingdom; Royal London Hospital, Barts Health NHS Trust, London, E1 1BB, United Kingdom; Department of Trauma and Orthopaedics, Southmead Hospital, North Bristol NHS Trust, Bristol, BS10 5NB, United Kingdom; Clinical & Operations Directorate, Royal Osteoporosis Society, Bath, BA2 3BH, United Kingdom; Musculoskeletal Research Unit, Translational Health Sciences, Bristol Medical School, University of Bristol, Bristol, BS10 5NB, United Kingdom; Musculoskeletal Research Unit, Translational Health Sciences, Bristol Medical School, University of Bristol, Bristol, BS10 5NB, United Kingdom; Population Health Sciences, Bristol Medical School, University of Bristol, Bristol, BS8 2PS, United Kingdom; Musculoskeletal Research Unit, Translational Health Sciences, Bristol Medical School, University of Bristol, Bristol, BS10 5NB, United Kingdom; Older People’s Unit, Royal United Hospital NHS Foundation Trust Bath, Combe Park, Bath, BA1 3NG, United Kingdom

**Keywords:** fragility fracture, fracture prevention, health services research, osteoporosis, refracture

## Abstract

Patients who sustain a hip fracture are known to be at imminent refracture risk. Their complex multidisciplinary rehabilitation needs to include falls prevention and anti-osteoporosis medication (AOM) to prevent such fractures. This study aimed to determine which hospital-level organizational factors predict prescription of post-hip fracture AOM and refracture risk. A cohort of 178 757 patients aged ≥60 yr who sustained a hip fracture in England and Wales (2016-2019) was examined and followed for 1 yr. Patient-level hospital admission datasets from 172 hospitals, the National Hip Fracture Database, and mortality data were linked to 71 metrics extracted from 18 hospital-level organizational reports. Multilevel models determined organizational factors, independent of patient case-mix, associated with (1) AOM prescription and (2) refracture (by ICD10 coding). Patients were mean (SD) 82.7 (8.6) yr old, 71% female, with 18% admitted from care homes. Overall, 101 735 (57%) were prescribed AOM during admission, while 50 354 (28%) died during 1-yr follow-up, 12 240 (7%) refractured. Twelve organizational factors were associated with AOM prescription, for example, orthogeriatrician-led care compared to traditional care models (odds ratio [OR] 4.65 [95% CI, 2.25–9.59]); AOM was 9% (95% CI, 6%–13%) more likely to be prescribed in hospitals providing routine bone health assessment to all patients. Refracture occurred at median 126 d (IQR 59–234). Eight organizational factors were associated with refracture risk; hospitals providing orthogeriatrician assessment to all patients within 72 h of admission had an 18% (95% CI, 2%–31%) lower refracture risk, weekend physiotherapy provision had an 8% (95% CI, 3%–14%) lower risk, and where occupational therapists attended clinical governance meetings, a 7% (95% CI, 2%–12%) lower risk. Delays initiating post-discharge community rehabilitation were associated with a 15% (95% CI, 3%–29%) greater refracture risk. These novel, national findings highlight the importance of orthogeriatrician, physiotherapist, and occupational therapist involvement in secondary fracture prevention post hip fracture; notably, fracture risk reductions were seen within 12 mo of hip fracture.

## Introduction

Incident fragility fractures accounted for €36.3 billion in annual direct medical costs in Europe in 2019; 57% due to hip fractures.^1^ Each year in the United Kingdom (UK), approximately 75 000 older adults sustain a fragility fracture of the hip, conveying a 28% 1-yr mortality^2–7^ Patients who sustain a hip fracture have a high imminent risk of refracture.^8^ Of those who present with a hip fracture, up to half have experienced a prior fragility fracture,^9^ 12% of those surviving a hip fracture go on to have another fracture within a year,^8^^,^^10^ and 25% within 5 yr.^11^ In contrast, osteoporosis treatments rates are low; in the UK, just 12% of patients presenting with a hip fracture are on established therapeutics, with the most recent national audit reporting only 51% are discharged from hospital on anti-osteoporosis medicine (AOM) to reduce future fracture risk.^12^ Furthermore, the proportion prescribed AOM during their admission varies markedly from 0% to 100% between hospitals in the UK, certainly more so than can be explained by patient case-mix, suggesting wide variation in clinical practice and the so called “postcode lottery” of health care, representing geographic variation in access to healthcare determined by the chance of where one lives.^13^

Patients who sustain a hip fracture require complex multidisciplinary care, testing organizational structures within hospitals.^14–16^ A second hip fracture is associated with even worse outcomes including higher mortality.^17^ Well organized and well-resourced healthcare services are needed to prevent refractures, including mechanisms by which to systematically prescribe AOM to reduce fracture risk.^14–16^ After a fragility fracture, prompt AOM treatment is essential as refracture risk is highest immediately after the fracture, and AOMs take time to lower fracture risk. Hence, there is an advantage to initiating treatment before hospital discharge and not delaying decision making waiting for—sometimes lengthy and difficult to access—outpatient follow-up.^18^ Falls risk assessment is equally imperative, with interventions to reduce falls risk also preventative of future fracture risk.^19^^,^^20^

This study aimed to determine, among patients with a hip fracture, which hospital-level organizational factors are associated with (1) anti-osteoporosis treatment prescription by the time of discharge from hospital and (2) refracture in the year following hip fracture, in England and Wales. An understanding of these factors will indicate areas on which to focus to improve hip fracture care delivery with the aim of reducing further fractures.

## Materials and methods

### Study population

The REDUCE study (REducing unwarranted variation in the Delivery of high qUality hip fraCture services in England and Wales) examined all index hip fracture cases (ie, first occurrence of hip fracture in the study period), in residents of England or Wales, aged 60 yr or older, and admitted to one of the 172 English or Welsh hospitals during the study period April 1, 2016 to March 31, 2019.^21^ Patients were followed-up for 12 mo post hip fracture, with the last follow-up date being March 31, 2020. Anonymized patient-level data were obtained from the routinely collected Hospital Episodes Statistics (HES) Admitted Patient Care database in England, and its Welsh equivalent the Patient Episode Database for Wales (PEDW). These data were linked by National Health Service (NHS) Digital to Office for National Statistics (ONS) Civil Registration Deaths data, and subsequently to data from the National Hip Fracture Database (NHFD), a national Healthcare Quality Improvement Partnership (HQIP) clinical audit of hip fracture care.

### Patient outcomes

#### Prescription of anti-osteoporosis medication

AOM use was coded as a binary variable based on treatments (including antiresorptive or osteoanabolic medicines) routinely recorded during the hospital stay in the NHFD (Table 1). AOM was coded as prescribed if the patient had an injectable or oral AOM either continued from pre-admission or started during the admission (Table 2). AOM was coded as absent if: (1) the patient was assessed and no AOM was considered to be needed or appropriate; (2) no assessment or action was taken; or (3) no AOM was prescribed as the patient was discharged to await a DXA scan or an outpatient appointment with an osteoporosis specialist. The national clinical audit only records antiresorptive or osteoanabolic treatment, calcium and/or vitamin D supplementation are not considered sufficient as secondary prevention in this patient group and are not recorded.

**Table 1:** Characteristics of patients admitted with a hip fracture to one of 172 hospitals in England and Wales, from 2016 to 2019, by anti-osteoporosis medication prescription during hospital admission and by refracture within the year post hip fracture.

		Anti-osteoporosis medication (AOM) recorded during hospital stay (*N* = 178 470)^a^					Refractures in the year post index hip fracture (*N* = 178 757)					Total	
		**AOM prescribed**		**No AOM prescribed**			**No refracture**		**Refracture**				
		***N***	**%**	***N***	**%**	***P*-value**	***N***	**%**	***N***	**%**	***P*-value**	***N***	**%**
**Total**	Row%	101 735	57	76 735	43		166 517	93	12 240	7		178 757	100
**Pre-fracture characteristics**													
**Age (yr)**	Mean (SD)	83.9	(7.5)	81.1	(9.7)		82.7	(8.7)	83.3	(8.1)		82.7	(8.6)
**Age (yr)**	60–69	4785	5	11 249	15		15 156	9	906	7		16 062	9
	70–79	20 834	20	20 201	26		38 596	23	2500	20		41 096	23
	80–89	52 237	51	28 495	37		74 840	45	6023	49		80 863	45
	90+	23 879	23	16 790	22	<.001	37 925	23	2811	23	<.001	40 736	23
**Sex**	Female	76 074	75	50 006	65	<.001	117 109	70	9169	75	<.001	126 278	71
**ASA grade** ^b^	I and II	25 264	25	19 911	26		42 602	26	2620	21		45 222	25
	III	61 130	60	41 041	53		94 696	57	7627	62		102 323	57
	IV and V	15 341	15	15 783	21	<.001	29 219	18	1993	16	<.001	31 212	17
**Hip fracture type**	Intracapsular	59 343	58	45 572	59		98 134	59	6948	57		105 082	59
	Inter, subtrochanteric, or other	42 392	42	31 163	41	<.001	68 383	41	5292	43	<.001	73 675	41
**Pre-fracture residence**	Own home/sheltered housing	84 563	83	61 843	81		136 197	82	10 445	85		146 642	82
	Not from own home	17 172	17	14 892	19	<.001	30 320	18	1795	15	<.001	32 115	18
**Pre-fracture mobility**	Freely mobile without walking aids	36 584	36	29 766	39		62 449	38	3991	33		66 440	37
	Mobile outdoors with 1 or 2 aids or frame	40 985	40	25 422	33		61 336	37	5185	42		66 521	37
	Some indoor, or no functional, mobility	24 166	24	21 547	28	<.001	42 732	26	3064	25	<.001	45 796	26
**Country**	England	97 018	95	71 133	93		156 984	94	11 375	93		168 359	94
	Wales	4717	5	5602	7	<.001	9533	6	865	7	<.001	10 398	6
**Outcomes**													
**Died at (d)**	7	1149	1	3140	4	<.001	–	–	–	–	<.001	4350	2
	30	4604	5	8434	11	<.001	–	–	–	–	<.001	13 126	7
	120	14 191	14	17 347	23	<.001	31 095	19	564	5	<.001	31 662	18
	365	24 863	24	25 339	33	<.001	47 926	29	2428	20	<.001	50 354	28
**Readmission by (d)**	30	15 104	15	10 116	13	<.001	22 098	13	3143	26	<.001	25 241	14
**Refracture by (d)**	365	7406	7	4812	6	<.001	–	–	–	–	<.001	12 240	7

a Excluded 287 without anti-osteoporosis medication data

b ASA, American Society of Anesthesiologists' grade: I and II (healthy patient or patient with mild systemic disease); III (patient with a severe but not incapacitating systemic disease); IV and V (a patient with an incapacitating disease that is also life-threatening or a moribund patient not expected to live for 24 h with or without surgery)

% represents column percentages unless stated otherwise

(Subsequent case-mix adjusted models include age group, sex, ASA grade, hip fracture type, pre-fracture residence, and pre-fracture mobility.)

**Table 2:** Characteristics of patients according to the reasons for not receiving/receiving anti-osteoporosis medication (AOM) (N = 178 470)^a^.

		Characteristics of those patients not on AOM treatment							
		**Assessed—no AOM needed/appropriate**			**No assessment or action taken**		**On no treatment - pending DXA scan or osteoporosis clinic assessment**		
		***N***	**%**		***N***	**%**	***N***	**%**	
**Total**	Row%	38 376	22		5518	3	32 841	18	
**Pre-fracture characteristics**									
**Age (yr)**	Mean (SD)	85.1	(8.5)		82.5	(9.0)	76.2	(9.0)	
**Age (yr)**	60–69	2288	6		566	10	8395	26	
	70–79	6476	17		1276	23	12 449	38	
	80–89	16 809	44		2403	44	9283	28	
	90+	12 803	33		1273	23	2714	8	
**Sex**	Female	25 323	66		3635	66	21 048	64	
**ASA grade** ^b^	I and II	5621	15		1326	24	12 964	39	
	III	21 900	57		2850	52	16 291	50	
	IV and V	10 855	28		1342	24	3586	11	
**Hip fracture type**	Intracapsular	21 940	57		3331	60	20 301	62	
	Inter, subtrochanteric, or other	16 436	43		2187	40	12 540	38	
**Pre-fracture residence**	Own home/sheltered housing	26 897	70		4449	81	30 497	93	
	Not from own home	11 479	30		1069	19	2344	7	
**Pre-fracture mobility**	Freely mobile without walking aids	10 063	26		1906	35	17 797	54	
	Mobile outdoors with 1 or 2 aids or frame	13 614	35		2027	37	9781	30	
	Some indoor, or no functional, mobility	14 699	38		1585	29	5263	16	
**Outcomes**									
**Died at (d)**	7	1889	5		1030	19	221	1	
	30	5989	16		1583	29	862	3	
	120	12 610	33		2081	38	2656	8	
	365	17 924	47		2466	45	4949	15	
**Readmission by (d)**	30	5612	15		497	9	4007	12	
**Refracture by (d)**	365	2386	6		298	5	2128	7	
		Characteristics of those patients on AOM treatment							
		**Continued from pre-admission—oral medication**		**Continued from pre-admission—injectable medication**		**Started on this admission—oral medication**		**Started on this admission—injectable medication**	
		***N***	**%**	***N***	**%**	***N***	**%**	***N***	**%**
**Total**	Row%	10 908	6	1584	1	73 485	41	15 758	9
**Pre-fracture characteristics**									
**Age (yr)**	Mean (SD)	83.3	(7.9)	82.0	(8.1)	83.8	(7.5)	85.2	(7.1)
**Age (yr)**	60–69	706	6	134	8	3467	5	478	3
	70–79	2388	22	412	26	15 426	21	2608	17
	80–89	5372	49	749	47	37 986	52	8130	52
	90+	2442	22	289	18	16 606	23	4542	29
**Sex**	Female	9146	84	1320	83	53 801	73	11 807	75
**ASA grade** ^b^	I and II	2487	23	341	22	19 527	27	2909	18
	III	6601	61	983	62	43 755	60	9791	62
	IV and V	1820	17	260	16	10 203	14	3058	19
**Hip fracture type**	Intracapsular	5550	51	793	50	44 034	60	8966	57
	Inter, subtrochanteric, or other	5358	49	791	50	29 451	40	6792	43
**Pre-fracture residence**	Own home/sheltered housing	8725	80	1369	86	62 016	84	12 453	79
	Not from own home	2183	20	215	14	11 469	16	3305	21
**Pre-fracture mobility**	Freely mobile without walking aids	3247	30	474	30	28 429	39	4434	28
	Mobile outdoors with 1 or 2 aids or frame	4609	42	666	42	29 478	40	6232	40
	Some indoor, or no functional, mobility	3052	28	444	28	15 578	21	5092	32
**Outcomes**									
**Died at (d)**	7	154	1	16	1	830	1	149	1
	30	540	5	68	4	3227	4	769	5
	120	1540	14	197	12	9904	13	2550	16
	365	2729	25	359	23	17 277	24	4498	29
**Readmission by (d)**	30	1707	16	236	15	10 706	15	2455	16
**Refracture by (d)**	365	917	8	154	10	5086	7	1249	8

a Excluded 287 without AOM data

b ASA, American Society of Anesthesiologists' grade: I and II (healthy patient or patient with mild systemic disease); III (patient with a severe but not incapacitating systemic disease); IV and V (a patient with an incapacitating disease that is also life-threatening or a moribund patient not expected to live for 24 h with or without surgery)

% represents column percentages unless stated otherwise

(Subsequent case-mix adjusted models include age group, sex, ASA grade, hip fracture type, pre-fracture residence, and pre-fracture mobility.)

#### Refracture

Refracture was defined using the primary diagnosis field (diag_01) in HES/PEDW admitted patient care datasets, which were searched for suitable International Classification of Diseases 10th edition, ICD10 codes (Table S1), occurring during hospital admissions more than 30 d and up to 1 yr after the index hip fracture admission date.^22^ A 30-d wash-out period was used to avoid double counting of admission fractures of the hip, and/or associated sites, and peri-operative periprosthetic fractures (PPFs) as a direct complication of surgery. Appropriate thresholds were investigated, and 30 d was chosen as the most conservative period, as it was reasoned that any organizational factor associated with refracture risk over 1 yr would be expected to convey an effect detectable beyond 30 d. Fractures of the hand, fingers, foot, toes, face, nose, and skull were excluded (as these fractures are not considered to be osteoporotic); fractures of the wrist, humerus, tibia, ankle, spine (including cervical spine), sternum, and ribs were included (as non-hip fractures), as well as second hip fractures (beyond 30 d). ICD10 fracture codes that included the word “pathological” were retained, as in NHS coding practice this can relate to the pathology of osteoporosis. Given the potential contribution of osteoporosis and falls to their mechanism, “late” (ie, beyond 30 d from operation) PPFs were also included.^23^^,^^24^

#### Patient case-mix

Case-mix adjustment was the same as that used in the NHFD clinical audit (Table 1),^25^ and included age, sex, American Society of Anesthesiologists’ (ASA) classification of pre-operative physical status,^26^ hip fracture type, pre-fracture residence, and pre-fracture mobility.^25^

#### Organizational-level data

Organizational factors potentially associated with patient outcomes were categorized using a systematic approach, as previously described^21^^,^^27^ and described below. National audits, data series, and ratings provide much publicly available data at the hospital provider level.^21^ Data were extracted from 18 such sources to characterize each component of the hip fracture care pathway from admission to discharge. Each data item (factor) was then mapped to one or more patient outcomes by expert consensus (expert reviewers were C.L.G., J.G., M.K.J., Y.B.-S., A.Jo., and T.C.),^21^ and each factor was assigned to one overarching theme (pre-, peri-, post-op, governance, or workload). This generated 71 relevant organizational factors (47 for AOM and 66 for refracture), which reflect care delivery throughout the hip fracture care pathway for bone health.^21^ Those organizational-level factors that were time-specific, were linked to patient-level data using hospital codes and the year (and month/quarter if available) corresponding to the date of hip fracture admission.

### Patient and public involvement and engagement

The REDUCE Study Patient and public involvement and engagement group comprised 4 individuals with osteoporosis and/or a history of hip fracture. This group contributed to the REDUCE ethics application, study design, materials and analysis approach, and their responses to a prioritization exercise of key hip fracture care organizational domains and patient outcomes informed this analysis.

### Approvals

Approvals were obtained from: NHS Health Research Authority, London City & East Research Ethics Committee (20/LO/0101); Royal College of Physicians Falls and Fragility Fracture Audit Programme (FFFAP/2018/003) and Healthcare Quality Improvement Partnership (HQIP330); NHS Wales Informatics Service (NWIS/30941); with an NHS Digital Data Sharing Agreement (DARS-NIC-334549-B1Y6X-v1.4).

### Statistical analysis

Patient case-mix characteristics were analyzed in a descriptive manner using frequency tabulations and summary statistics by outcome groups. Chi-squared (χ^2^) tests were used to assess associations between categorical variables. Multilevel logistic regression models were used to estimate associations between organizational-level factors and patient-level outcomes, adjusting for patient case-mix. The hierarchical data structure consisted of patients (level 1) nested within hospitals (level 2). Depending on the format of the original data, organizational factors were used in their available categories, only combining categories where numbers were very small, to give dichotomized or categorized factors. If organizational factors were continuous, they were converted to linear splines at quartiles (or tertiles when data did not lend itself to quartiles). Backward stepwise elimination identified the organizational factors most strongly associated with each outcome. The identified organizational factors were further simplified by expert review, and splines were dichotomized, categorized, or converted back to continuous measures as indicated by the stepwise regression. The assumption of the multi-level model was linearity of continuous organizational factors with outcome, and linear splines were used to check this assumption. If the association was non-linear, then categories were combined or continuous data cut at thresholds indicated by the models to give the simplest groupings. If the association was linear, the organizational factors were included as a continuous measure on an appropriate scale. The organizational factors with the strongest evidence supporting associations with AOM and/or refracture outcomes, adjusted for case-mix, and mutually adjusted for all selected organizational factors are reported. Statistical analyses were performed in Stata version 16.1 and MLwiN version 3.01 (Centre for Multilevel Modelling, University of Bristol, Bristol, UK). STROBE (Strengthening the Reporting of Observational Studies in Epidemiology) guidance was followed throughout.^28^

## Results

### Study population

Between April 1, 2016 and March 31, 2019, 178 757 patients were identified with an index hip fracture from 172 hospitals with 168 to 2552 patients per hospital; 168 359 (94%) from England and 10 398 (6%) from Wales. Mean age was 82.7 yr (SD 8.6) in England and 82.3 yr (SD 8.6) in Wales, and 126 278 (71%) were women and 52 479 (29%) were men. In total, 82% were admitted from their own home/sheltered housing, as opposed to a care home. During the 3-yr study period, the 172 hospitals each admitted a median of 1026 (IQR 759–1282) patients with hip fracture, with an annual (in 2018-2019) mean of 355 (IQR 246–421) hip fracture admissions per hospital. All patients were followed up for 365 d post index hip fracture, or until death during this period. The median length of stay in hospital was 15 d (IQR 9–26), and the median period of follow-up was 365 d (IQR 276–365).

### Anti-osteoporosis medication

A total of 178 470 patients (>99% of 178 757 in the cohort) had AOM data recorded: AOM was continued from pre-admission in 7% (oral 6% or injectable 1%); AOM was started during the admission in 50% (oral 41% or injectable 9%); the patient was assessed, and no AOM was considered to be needed or appropriate in 22%; no assessment or action was taken in 3%; and no AOM was prescribed as the patient was discharged to await a DXA scan or an outpatient appointment in 18%.

In total, 101 735 (57%) patients were discharged with AOM; 12 492 (7%) were identified as continuing an AOM from pre-admission, and 89 243 (50%) as starting an AOM following hip fracture admission. Those prescribed AOM were more likely to be older (mean age 84 vs 81 yr), female (75% vs 65%), and to have better pre-fracture mobility (ie, freely mobile without walking aids or “mobile outdoors with 1 or 2 aids or frame”) (76% vs 72%) compared to those not prescribed AOM (Table 1). Across each of the 172 hospitals, the percentage of patients with hip fracture prescribed AOM by the time of discharge varied from 4% to >99%.

Overall, 32 841 patients (18%) were discharged to await an AOM treatment decision pending a DXA scan or an outpatient specialist assessment in an osteoporosis clinic; however, this percentage of hip fracture patients awaiting an AOM treatment decision ranged from 0% to 92% between hospitals (IQR 9%–26%). A greater proportion of younger patients was discharged pending a DXA scan or outpatient osteoporosis specialist assessment, with fewer waiting in the oldest age group, for example., 26% aged 60–69 vs 8% aged 90+ (Table 2), compared to the whole cohort where the age distribution was 9%–45% aged 60–89 and 23% aged 90+. Mortality rate was higher at all timepoints (ie, 7, 30, 120, and 365 d) for patients who were not assessed, or who were recorded as “inappropriate for AOM,” when compared to those referred for a DXA or osteoporosis clinic assessment (Table 2).

When limited to those in whom treatment had been continued or initiated or decided against or with no action taken (ie, excluding those pending further investigation) by the time of discharge (*n* = 145 629), age was associated with AOM treatment. Those aged 70–89 yr were most likely to be treated with an AOM (73% treated), while this was less likely (63%) among younger patients aged 60–69 yr, or among those aged 90+. Women were more likely to be prescribed AOM than men (72% among women vs 63% among men).

**Figure 1:**
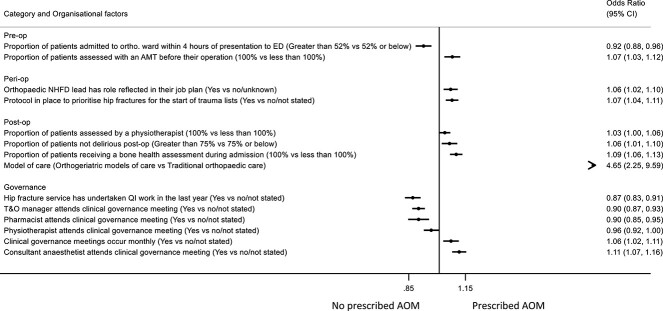
The association between organizational factors and anti-osteoporosis medication (AOM) prescription, accounting for patient case-mix and other organizational factors (*N* = 178 470). OR > 1 indicates more likely to have anti-osteoporosis medicine (AOM) prescribed. Organizational factors adjusted for case-mix (age group, sex, ASA classification, hip fracture type, pre-fracture residence, and pre-fracture mobility) and mutually adjusted for all backward selected factors shown in Table S2. Factors with *P*-value<0.1 shown. AMT, abbreviated mental test; ASA, American Society of Anesthesiologists; CI, confidence interval; ED, emergency department; NHFD, National Hip Fracture Database; op, operative; OR, odds ratio; QI, quality improvement; T&O, trauma and orthopedics.

Twelve organizational factors were associated with AOM treatment (Figure 1**,**  Tables S2 and S3). Data sources are listed in Table S2**,** for example, NHFD facilities audit, Best Practice and Key Performance Indicators (KPIs), and frequencies of organizational factors by the different AOM categories are provided in Table S3. The results were dominated by a very strong association between the model of hip fracture care in use; hospitals with an orthogeriatrician-led care model were much more likely to prescribe AOM compared to hospitals with traditional orthopedic-led models (OR 4.65 [95% CI, 2.25–9.59]). However, few hospitals (3%) reported a traditional model of orthopedic care vs 97% with an orthogeriatric model of care; such orthogeriatric models include shared care/admitted under geriatricians (46%), routine orthogeriatric review (36%), post-operative geriatric care (6%), or another model of care (9%).

Hospitals routinely assessing cognitive function pre-operatively in all patients, using the Abbreviated Mental Test (AMT), were 7% more likely to discharge patients on AOM [95% CI, 3%–12%]. Hospitals where fewer than 25% of patients were delirious post-operatively were more likely to discharge patients on AOM (6% [95% CI,1%–10%]), as were those hospitals where all patients received a bone health assessment during their admission (9% [95% CI, 6%–13%]). Hospitals where the orthopedic NHFD lead role was reflected in their job plan, or where there was a protocol in place to prioritize hip fractures at the start of trauma lists, were more likely to discharge patients on AOM (6% [95% CI, 2%–10%] and 7% [95% CI, 4%–11%], respectively). In contrast, hospitals where patients were admitted to an orthopedic ward within 4 h of presentation to the emergency department were less likely to receive AOM (−8% [95% CI, −4% to −12%]).

In terms of governance, hospitals where clinical governance meetings occurred monthly and where these meetings were attended by a consultant anesthetist, were more likely to discharge patients on AOM (6% [95% CI, 2%–11%] and 11% [95% CI, 7%–16%], respectively). Conversely, attendance by departmental managers and pharmacists was associated with lower use of AOM on discharge (−10% [95% CI, −7% to −13%] and −10% [95% CI, −5% to −15%], respectively). Furthermore, those hospitals with hip fracture services undertaking quality improvement work were less likely to discharge patients on AOM (−13% [95% CI, −9% to −17 %]).

### Sensitivity analysis

Initiation of AOM may be considered inappropriate for some patients, for example, those in the last weeks to months of life or with end stage renal failure; hence, AOM use was examined against mortality at 120 d. In 178 470 with available data, a greater number of patients discharged without AOM had died by 120 d compared to those discharged with AOM (120-d mortality 23% vs 14%; *P*<.001), findings were amplified after excluding those awaiting DXA scans or osteoporosis clinic appointments (120-d mortality 33% vs 14%; *P*<.001. *N* = 145 629). Comparatively, 14% of patients who were discharged on AOM had died by 120 d. Of those not discharged with AOM, the highest mortality was seen in those where the NHFD had recorded “No assessment or action taken” (120-d mortality 38% [2081/5518]) and those “Assessed – no AOM needed/appropriate” (120-d mortality 33% [12 610/38 376]), compared to a 120-mortality of 8% [2656/32 841] in the group “On no treatment – pending DXA scan or osteoporosis clinic assessment” Table 2).

Hence, in a sensitivity analysis, models were re-run excluding the 32 841 patients recorded as “On no treatment – pending DXA scan or osteoporosis clinic assessment,” which had been included in the “no AOM on discharge” group. Of the 12 associated organizational factors, the majority remained consistent in direction and effect size. Two associations were attenuated so that confidence intervals crossed the null and *P*-values ≥.1: (1) pre-operative AMT assessment (OR 1.03 [95% CI, 0.98–1.08] *P*=.296 in sensitivity analysis) and (2) peri-operative hip fracture trauma list prioritization (OR: 1.03 [95% CI, 0.99–1.07] *P*=.118). Findings were then similar after the further exclusion of those 5518 patients with “No assessment or action taken” during admission. Additional sensitivity analysis classified those with “no AOM was prescribed as the patient was discharged to await a DXA scan or an outpatient appointment” as AOM treatment prescribed, resulting in 134 576 patients coded as AOM prescribed and 43 894 coded as no AOM prescribed. Findings were again similar to those mentioned above with the same 2 organizational factors (pre-operative AMT assessment and peri-operative hip fracture trauma list prioritization) attenuated so that confidence intervals crossed the null. Findings were also substantively similar if the 29 hospitals with more than 30% of patients waiting for an assessment were excluded from the analysis (92 360 patients coded as AOM prescribed and 57 586 coded as no AOM prescribed).

### Refracture

**Figure 2:**
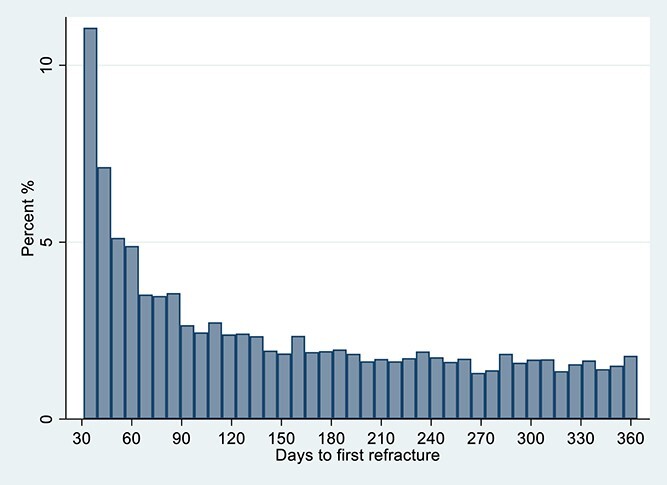
Days to refracture from 30 d and up to 365 d post hip fracture admission *N* = 12 240.

In total, 12 240 (7%) sustained a further fracture in the year after their index hip fracture admission; across the 172 hospitals, the percentage of patients who refractured ranged from 4% to 13%. Those patients with refracture tended to be older than those refracture-free (mean age 83.3 vs 82.7 yr), more likely to be female (75 vs 70%) and were less mobile pre-index hip fracture (33 vs 38% freely mobile without walking aids) (Table 1). The median number of days to the first refracture was 126 d (IQR 59–234 d) (Figure 2). During the 1 yr of follow-up, 50 354 patients (28%) died.

**Figure 3:**
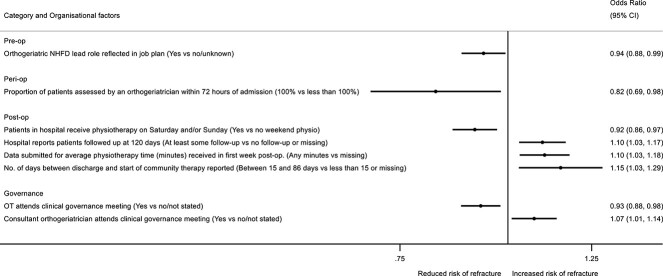
The association between organizational factors and refracture in the year post hip fracture, accounting for patient case-mix and other organizational factors. *N* = 178 757, OR > 1 indicates increased risk of refracture. Organizational factors adjusted for case-mix (age group, sex, ASA classification, hip fracture type, pre-fracture residence, and pre-fracture mobility) and mutually adjusted for all backward selected factors shown in Table S4. Factors with *P*-value<.1 shown. ASA, American Society of Anesthesiologists; CI, confidence interval; NHFD, National Hip Fracture Database; op, operative; OR, odds ratio; OT, occupational therapist; QI, quality improvement; T&O, trauma and orthopedics.

Eight organizational factors were independently associated with patient refracture risk (Figure 3**,**  Tables S4 and S5). Hospitals providing orthogeriatrician assessment to all patients within 72 h of admission had an 18% (95% CI, 2%–31%) lower risk of refracture among their patients. In addition, those with orthogeriatric leadership roles reflected in job plans had a 6% (95% CI, 1%–12%) lower refracture risk. Weekend delivery of physiotherapy was associated with a further 8% (95% CI, 3%–14%) lower refracture risk. Conversely, hospitals discharging patients to the community where patients waited more than 14 d (up to 86 d) before community-based physiotherapy started, saw higher refracture risk (15% [95% CI, 3%–29%]), compared to hospitals discharging to more prompt community therapy services or with no submitted data. Hospitals with more complete data for some measures were also those with higher detected refracture risk, for example hospitals reporting patients followed up at 120 d (10% [95% CI, 3%–17%]), as were those that had data concerning the amount of physiotherapy received in the first inpatient week (10% [95% CI, 3%–18%]).

In terms of governance, participation by occupational therapists in clinical governance meetings was associated with a 7% (95% CI, 2%–12%) reduced risk of refracture. However, hospitals where a consultant orthogeriatrician routinely attended clinical governance meetings had greater refracture risks among their patients (7% [95% CI, 1%–14%]).

Of those discharged without AOM, there was a lower refracture rate of 6% [4812/76 735] compared to those discharged with AOM (refracture of 7% [7406/101 735] χ^2^  *P*<.001) (Table 1). The proportion of patients experiencing a refracture for each category of AOM treatment was: AOM continued from pre-admission, oral (8%), or injectable (10%); AOM started during the admission, oral (7%), or injectable (8%); patient assessed and no AOM considered to be needed or appropriate (6%); no assessment or action was taken (5%); or no AOM was prescribed as the patient was discharged to await a DXA scan or an outpatient appointment (7%) (Table 2).

### Sensitivity analysis

In a sensitivity analysis, the final refracture model was re-run to include only the 128 403 patients recorded as alive at 365 d. The association between refracture and organizational factors was similar to those reported above. To examine the possibility that index fractures had been misclassified as refractures using the 30 d cut off, a sensitivity analysis was conducted omitting those with refractures before 60 d (*N* = 3121), which produced similar findings.

## Discussion

In England and Wales, the percentage of patients starting anti-osteoporosis medication following an admission to hospital for hip fracture is 57%. Twelve organizational factors were associated with AOM treatment by the time of hospital discharge. The strongest association was seen for orthogeriatric models of inpatient hip fracture care, such that hospitals with this model were more than 4 times more likely to discharge patients on AOMs, compared to hospitals still providing traditional models of orthopedic-led care. AOM was 9% (95% CI, 6%–13%) more likely to be prescribed in hospitals providing routine bone health assessment to all patients. Eight organizational factors were associated with refracture risk; hospitals providing orthogeriatrician assessment to all patients within 72 h of admission had an 18% (95% CI, 2%–31%) lower refracture risk, weekend physiotherapy provision an 8% (95% CI, 3%–14%) lower risk, and where occupational therapists attended clinical governance meetings, a 7% (95% CI, 2%–12%) lower risk. Delays initiating post-discharge community rehabilitation were associated with a 15% (95% CI, 3%–29%) greater refracture risk.

Discharge on AOM was (9%) more likely in hospitals where all patients received a routine bone health assessment during their admission; this is understandable as this is an assessment largely performed by orthogeriatricians. Furthermore, hospitals providing orthogeriatrician assessment to all patients within 72 h of admission had an 18% reduced refracture risk in the year following hip fracture among their patients. However, AOM treatment is only one part of fracture prevention: almost all fragility fractures result from a fall, and a key aim of post-hip fracture rehabilitation is to restore safe mobility. Hence, it is notable that the study found provision of inpatient weekend physiotherapy to be associated with 8% reduced refracture risk. The role of occupational therapists (OTs) in home safety assessment and coordination of safe discharges may well explain why units in which OTs attended the hip fracture clinical governance meetings recorded 7% reduced risk of refracture. Importantly, delays of more than 2 wk in initiating post-discharge community therapy were associated with a substantial 15% increase in refracture risk.

Only 7% of patients were already on AOM when they presented with their index hip fracture. This study defined treatment with AOM as leaving hospital either continuing any pre-admission AOM or having started treatment before discharge, this did not include those referred for initiation of treatment in the community or clinic. Hence, differences in AOM prescribing may reflect local prescribing practices, as some hospitals will hand responsibility for AOM initiation to a local fracture liaison service, which will start treatment after the patient has left hospital, although delays in this pathway are well documented.^12^ Not all patients will be suitable for AOM, for some it may be inappropriate because they are terminally ill or have severe renal failure. As expected, a greater proportion of patients discharged without AOM died by 120 d compared to those discharged with AOM^29^; however, 6% (*n* = 2386) of patients who were assessed and considered not to need AOM or in whom AOM treatment would be inappropriate went on to refracture within 12 mo—a similar proportion to those who were chosen to receive an oral AOM (7%), suggesting widespread undertreatment. Of course initiating an AOM may not have prevented refracture given the length of time needed for AOMs to reduce risk. However, therapeutic nihilism is well known following hip fracture,^30^ in part due to the well-recognized significant 1 yr mortality. Our findings show that even among those who die within a year of hip fracture, 1 in 20 refracture before they die. Together, these findings suggest that AOM treatment may still provide a clinical benefit to many currently denied treatment, potentially preventing a painful event +/− death. Notably, the median length of stay in hospital (acute + rehabilitation combined) was 15 d in this cohort, sufficient time to administer a first dose of intravenous zoledronate^31^^,^^32^; however, concerningly, only 9% of patients received this AOM.^33^ Systematic prescription of AOM to reduce secondary fracture risk is increasingly important given population ageing and the rising numbers with age-associated fragility fractures. If greater use of intravenous zoledronate can be achieved, as has recently been recommended,^31^ future research might determine the organizational factors associated with successful treatment delivery and re-fracture risk reduction.

Unsurprisingly, hospitals where all patients were reported to receive a bone health assessment, discharged more patients on AOM. A systematic review and meta-analysis found that orthogeriatric care models were more likely to discharge patients on AOM than traditional orthopedic models of care, but found the effect of orthogeriatric care on refracture to be inconclusive.^34^ Our study extends this, as it has shown both that orthogeriatric care models are the strongest predictor of effective provision of AOM, and importantly that it was an orthogeriatric assessment within 72 h of admission, rather than the care model used to describe the service that was most strongly associated with reduced refracture risk.

AOM was more likely to be prescribed in hospitals where the orthopedic NHFD lead role was reflected in their job plan, where a protocol was in place to prioritize hip fractures for the start of surgical trauma operating lists, where all pre-operative patients had an AMT, where post-op delirium was less common and where clinical governance meetings occurred monthly and had a consultant anesthetist attending. These observations all suggest that well-organized hospitals, with established protocols and better patient care, are also better able to discharge patients on AOM.

Perhaps surprisingly, we found that lower use of AOM is seen in hospitals where department managers and pharmacists attend clinical governance meetings, and where departmental quality improvement work had recently been conducted. This may be due to reverse causality, whereby problems in assessing bone health and/or prescribing medicines mean managers and/or pharmacists are called to attend governance meetings to address problems with service delivery, and whereby improvement work may be needed to address problems within the hip fracture services. Alternatively, these organizational factors may result in more patients being referred for outpatient DXA for a more personalized risk-based approach to AOM prescribing.

In hospitals where more patients were admitted to orthopedic wards within 4 h, there was a lower likelihood of AOM treatment. This may indicate the lack of a hip fracture unit in the hospital, with patients admitted directly to a standard trauma and orthopedic ward, where there is no orthogeriatric service and thus they are not prescribed AOMs; the data appear to support this. Equally, large, busy hospitals with more patients may have less time to prescribe AOM as patients are prioritized for surgery and rapidly moved through departments, so there is less time for comprehensive assessment and treatment. Alternatively, if a shorter length of stay is seen in those not prescribed AOM this may suggest that patients pass through the acute hospital quickly and are discharged home or to a rehabilitation facility without AOM, but with a plan for follow-up assessment and prescription instead, after a DXA scan or an outpatient appointment. Of the 18% patients not treated with AOM, pending a DXA scan or an outpatient appointment 7% went on to refracture within 12 mo, raising the possibility that had a prompt treatment decision been made to initiate AOM, this refracture rate may have been lower.

Refracture was most common immediately after the index hip fracture, consistent with the growing literature on high imminent refracture risk.^18^^,^^35^ Orthogeriatrician assessment within 72 h of admission was the strongest predictor of reduced 1-yr refracture risk and is a NHFD KPI, as well as being one of the criteria for NHS England’s financial incentive of Best Practice Tariff (BPT), which has driven up provision of orthogeriatrician assessment since being recognized as highly cost-effective.^15^ This finding is supported by the association with the orthogeriatrician lead role reflected in job planning. Conversely, orthogeriatricians’ attendance at clinical governance meetings was associated with a higher rate of refracture—an unexpected, perhaps chance, finding which cannot be explained by the data available. Higher refracture rates were associated with post-discharge follow-up at 120 d and submission of detailed data regarding inpatient physiotherapy provision. These measures may be collected to address specific issues identified within a hip fracture service to inform quality improvement initiatives aimed at addressing problems in the service.

The Physiotherapy Hip Fracture Sprint Audit^36^ identified wide variation in how well different hospital teams coordinate discharge and post-discharge care with community rehabilitation teams, and how long patients had to wait for such rehabilitation to start after their return home. In this study, delays of more than 2 wk in initiating community rehabilitation therapy were associated with a 15% increase in refracture risk, and in previous work with increased days spent in hospital and higher inpatient costs in the year post hip fracture.^37^ These findings are consistent with the benefits of hospitals providing a continuous service, whereby patient mobility and functional needs are met by streamlined transfer of care to community services to reduce fall and refracture risk.

This study has strengths; use of a unique linkage of national databases for NHS-treated patients across 2 nations, with 18 different organizational data sources. The 3-yr study period allowed for temporal fluctuations, giving representative overall estimates for each hospital. Multilevel analysis, accounting for within-hospital clustering, enabled a true hospital-level assessment of associations. Routinely collected data such as HES ICD10 coding have been shown to be improving in accuracy over time,^38^ and since hospital reimbursement is based on these data, a great deal of effort is invested in correctly assigning these diagnosis codes. Furthermore, both surgical hip fracture care and provision of bone assessment are specifically addressed in the national clinical audit, and both are financially incentivized through NHS England’s BPT, so correct coding of fracture location, timing, and type is of great importance. Limitations include that the study was unable to identify “early” refractures, occurring during the first 30 d after the index admission date, as it was not possible to differentiate the index from a subsequent fracture at the same or contralateral site using HES codes. Due to this washout period, which was selected to prevent misclassification of index hip fractures as refractures, not all refractures will have been captured, hence the outcome can be considered as “later refracture” in orthopedic terms. However, the study aimed to examine factors associated with prevention of osteoporotic refractures, so early refractures—potentially intra-operative fractures—in this perioperative period are less relevant, and it was considered that any organizational factor associated with refracture risk over 1 yr would be expected to convey an effect detectable beyond 30 d. The study lacked data on those patients who were offered but refused AOM treatment; those referred for AOM post discharge; adherence post discharge for those on oral AOM; and patient-reported outcomes, which were not available and could be addressed in future research. Overall, 18% of the cohort were not receiving AOM pending further assessment. DXA scans are requested either (1) to prove that a fracture was associated with bone fragility (the injury may have been unusually severe, or the patient unconvinced by suggestions of bone fragility; however, many such scans show normal bone density^39^), (2) to provide a baseline measurement for more intensive treatment options (for those judged to be at very high fracture risk), or (3) the unit passes all osteoporosis assessments on to an outpatient clinic. The true reason for referral for assessment is not captured in these datasets and is a limitation. However, sensitivity analyses indicated that the main findings were robust, when excluding this group, or when coding them as treated, or when excluding hospitals where more than 30% patients were awaiting assessment.

Large sample sizes can generate associations that appear important statistically, which may not be clinically meaningful and are prone to type 1 error. Causality cannot be inferred from these observational data and despite the use of multivariable models, there may still be hospital-level residual confounding. There is also the risk of the ecological fallacy, so that protective factors that operate at a hospital level may not apply at an individual level. However, many of these organizational factors are ecological in nature, for example, governance procedures, and would apply to all patients within a hospital. While these organizational factors were derived from high-quality NHFD and other publicly available audits and NHS data, these could not be independently validated. Some audits were troubled by missing data, such that some variables could not be used, meaning some components of the care pathway could not be operationalized and remain unmeasured. Some audits with fewer missing data required inclusion of a missing category or were supplemented with data from available years. Stepwise selection may detect coincidental associations while missing some causal associations; and multilevel models made bootstrapping too computationally intensive to provide internal validation. Since the exact time each patient started AOM is not available, and as patients must be alive to start AOM or have another fracture, the analysis is prone to “immortal time bias” and not adjusted for competing risk of death.

## Conclusions

This novel, national study highlights the importance of orthogeriatric care, physio- and occupational therapy provision in secondary fracture prevention in frail hip fracture populations, which may be generalizable to other countries. Fracture risk reductions associated with this multidisciplinary care provision are apparent within a year of hip fracture; notably, longer timeframes may be needed to see such fracture risk reductions from some anti-osteoporosis treatments.

## Supplementary Material

REDUCE_bone_refrac_v1_5_Suplementary_Materials_240415_v3a_zjae100

## Data Availability

Electronic health records are, by definition, considered sensitive data in the UK by the Data Protection Act and cannot be shared via public deposition because of information governance restriction in place to protect patient confidentiality. Access to data is available once approval has been obtained through the individual constituent entities controlling access to the data. Researchers interested in accessing: (1) HES data can apply for access through NHS Digital’s Data Access Request Service (DARS) https://dataaccessrequest.hscic.gov.uk/; (2) PEDW data can apply for access through the NHS Informatics Service—PEDW Data Online https://nwis.nhs.wales/information-services/health-intelligence/pedw-data-online/; and (3) NHFD data can apply for access through the Falls and Fragility Fracture Audit Programme (FFFAP) Expression of interest form https://www.rcplondon.ac.uk/file/fffap-expression-interest-form-1/ and a Data Access Request Form (DARF) https://www.rcplondon.ac.uk/guidelines-policy/applying-work-falls-and-fragility-fracture-audit-programme-data/.
