# Supplementary material for: Following hip fracture, hospital organizational factors associated with prescription of anti-osteoporosis medication on discharge, to address imminent refracture risk: a record-linkage study
Source: J Bone Miner Res. 2024 Jul 11;39(8):1071–82. doi: 10.1093/jbmr/zjae100 (PMC11337946; doi:10.1093/jbmr/zjae100)
Supplement: REDUCE_bone_refrac_v1_5_Suplementary_Materials_240415_v3a_zjae100 [file reduce_bone_refrac_v1_5_suplementary_materials_240415_v3a_zjae100.docx]

## Supplementary materials:

## Table S1: List of ICD10 codes to identify refracture

| ICD10 code | Description |
| --- | --- |
| M484 | Fatigue fracture of vertebra- |
| M485 | Collapsed vertebra, not elsewhere classified- |
| M800 | Postmenopausal osteoporosis with pathological fracture- |
| M8000 | Postmenopausal osteoporosis with pathological fracture-Multiple sites |
| M8001 | Postmenopausal osteoporosis with pathological fracture-Shoulder region |
| M8002 | Postmenopausal osteoporosis with pathological fracture-Upper arm |
| M8003 | Postmenopausal osteoporosis with pathological fracture-Forearm |
| M8005 | Postmenopausal osteoporosis with pathological fracture-Pelvic region and thigh |
| M8006 | Postmenopausal osteoporosis with pathological fracture-Lower leg |
| M8007 | Postmenopausal osteoporosis with pathological fracture-Ankle and foot |
| M8008 | Postmenopausal osteoporosis with pathological fracture-Other |
| M800AX | M80-Osteoporosis with pathological fracture |
| M801 | Postoophorectomy osteoporosis with pathological fracture- |
| M802 | Osteoporosis of disuse with pathological fracture- |
| M803 | Postsurgical malabsorption osteoporosis with pathological fracture- |
| M804 | Drug-induced osteoporosis with pathological fracture- |
| M805 | Idiopathic osteoporosis with pathological fracture- |
| M808 | Other osteoporosis with pathological fracture- |
| M8080 | Other osteoporosis with pathological fracture-Multiple sites |
| M8081 | Other osteoporosis with pathological fracture-Shoulder region |
| M8082 | Other osteoporosis with pathological fracture-Upper arm |
| M8083 | Other osteoporosis with pathological fracture-Forearm |
| M8085 | Other osteoporosis with pathological fracture-Pelvic region and thigh |
| M8086 | Other osteoporosis with pathological fracture-Lower leg |
| M8087 | Other osteoporosis with pathological fracture-Ankle and foot |
| M8088 | Other osteoporosis with pathological fracture-Other |
| M808AX | M80-Osteoporosis with pathological fracture |
| M809 | Unspecified osteoporosis with pathological fracture- |
| M8431 | Stress fracture, not elsewhere classified-Shoulder region |
| M8432 | Stress fracture, not elsewhere classified-Upper arm |
| M8433 | Stress fracture, not elsewhere classified-Forearm |
| M84350 | M84-Disorders of continuity of bone |
| M84351 | M84-Disorders of continuity of bone |
| M84352 | M84-Disorders of continuity of bone |
| M84353 | M84-Disorders of continuity of bone |
| M84359 | M84-Disorders of continuity of bone |
| M8436 | Stress fracture, not elsewhere classified-Lower leg |
| M84371 | M84-Disorders of continuity of bone |
| M84372 | M84-Disorders of continuity of bone |
| M84373 | M84-Disorders of continuity of bone |
| M8440 | Pathological fracture, not elsewhere classified-Multiple sites |
| M8440X | M84-Disorders of continuity of bone |
| M8441 | Pathological fracture, not elsewhere classified-Shoulder region |
| M8442 | Pathological fracture, not elsewhere classified-Upper arm |
| M8443 | Pathological fracture, not elsewhere classified-Forearm |
| M8445 | Pathological fracture, not elsewhere classified-Pelvic region and thigh |
| M84451 | M84-Disorders of continuity of bone |
| M84452 | M84-Disorders of continuity of bone |
| M84453 | M84-Disorders of continuity of bone |
| M84454 | M84-Disorders of continuity of bone |
| M84459 | M84-Disorders of continuity of bone |
| M8446 | Pathological fracture, not elsewhere classified-Lower leg |
| M84471 | M84-Disorders of continuity of bone |
| M84472 | M84-Disorders of continuity of bone |
| M84473 | M84-Disorders of continuity of bone |
| M8448 | Pathological fracture, not elsewhere classified-Other |
| M8448X | M84-Disorders of continuity of bone |
| M8450X | M84-Disorders of continuity of bone |
| M84511 | M84-Disorders of continuity of bone |
| M84512 | M84-Disorders of continuity of bone |
| M84519 | M84-Disorders of continuity of bone |
| M84521 | M84-Disorders of continuity of bone |
| M84522 | M84-Disorders of continuity of bone |
| M84529 | M84-Disorders of continuity of bone |
| M84531 | M84-Disorders of continuity of bone |
| M84532 | M84-Disorders of continuity of bone |
| M84533 | M84-Disorders of continuity of bone |
| M84534 | M84-Disorders of continuity of bone |
| M84539 | M84-Disorders of continuity of bone |
| M84550 | M84-Disorders of continuity of bone |
| M84551 | M84-Disorders of continuity of bone |
| M84552 | M84-Disorders of continuity of bone |
| M84553 | M84-Disorders of continuity of bone |
| M84559 | M84-Disorders of continuity of bone |
| M84561 | M84-Disorders of continuity of bone |
| M84562 | M84-Disorders of continuity of bone |
| M84563 | M84-Disorders of continuity of bone |
| M84564 | M84-Disorders of continuity of bone |
| M84569 | M84-Disorders of continuity of bone |
| M84571 | M84-Disorders of continuity of bone |
| M84572 | M84-Disorders of continuity of bone |
| M84573 | M84-Disorders of continuity of bone |
| M8458X | M84-Disorders of continuity of bone |
| M8460X | M84-Disorders of continuity of bone |
| M84611 | M84-Disorders of continuity of bone |
| M84612 | M84-Disorders of continuity of bone |
| M84619 | M84-Disorders of continuity of bone |
| M84621 | M84-Disorders of continuity of bone |
| M84622 | M84-Disorders of continuity of bone |
| M84629 | M84-Disorders of continuity of bone |
| M84631 | M84-Disorders of continuity of bone |
| M84632 | M84-Disorders of continuity of bone |
| M84633 | M84-Disorders of continuity of bone |
| M84634 | M84-Disorders of continuity of bone |
| M84639 | M84-Disorders of continuity of bone |
| M84650 | M84-Disorders of continuity of bone |
| M84651 | M84-Disorders of continuity of bone |
| M84652 | M84-Disorders of continuity of bone |
| M84653 | M84-Disorders of continuity of bone |
| M84659 | M84-Disorders of continuity of bone |
| M84661 | M84-Disorders of continuity of bone |
| M84662 | M84-Disorders of continuity of bone |
| M84663 | M84-Disorders of continuity of bone |
| M84664 | M84-Disorders of continuity of bone |
| M84669 | M84-Disorders of continuity of bone |
| M84671 | M84-Disorders of continuity of bone |
| M84672 | M84-Disorders of continuity of bone |
| M84673 | M84-Disorders of continuity of bone |
| M8468X | M84-Disorders of continuity of bone |
| M8475 | M84-Disorders of continuity of bone |
| M966 | Fracture of bone following insertion of orthopaedic implant, joint prosthesis, or bone plate- |
| M9662 | M96-Postprocedural musculoskeletal disorders, not elsewhere classified |
| M9663 | M96-Postprocedural musculoskeletal disorders, not elsewhere classified |
| M9665 | M96-Postprocedural musculoskeletal disorders, not elsewhere classified |
| M9666 | M96-Postprocedural musculoskeletal disorders, not elsewhere classified |
| M9667 | M96-Postprocedural musculoskeletal disorders, not elsewhere classified |
| M9669 | M96-Postprocedural musculoskeletal disorders, not elsewhere classified |
| M97 | Periprosthetic fracture around internal prosthetic hip joint |
| M970 | Periprosthetic fracture around internal prosthetic hip joint |
| M971 | Periprosthetic fracture around internal prosthetic hip joint |
| M972 | Periprosthetic fracture around internal prosthetic hip joint |
| M973 | Periprosthetic fracture around internal prosthetic hip joint |
| M974 | Periprosthetic fracture around internal prosthetic hip joint |
| M978 | Periprosthetic fracture around internal prosthetic hip joint |
| M979 | Periprosthetic fracture around unspecified internal prosthetic joint |
| S120 | Fracture of first cervical vertebra- |
| S121 | Fracture of second cervical vertebra- |
| S122 | Fracture of other specified cervical vertebra- |
| S123 | S12-Fracture of neck |
| S124 | S12-Fracture of neck |
| S125 | S12-Fracture of neck |
| S126 | S12-Fracture of neck |
| S127 | Multiple fractures of cervical spine- |
| S128 | S12-Fracture of neck |
| S129 | Fracture of neck, part unspecified- |
| S1290 | Fracture of neck, part unspecified-closed |
| S129XX | S12-Fracture of neck |
| S220 | Fracture of thoracic vertebra- |
| S221 | Multiple fractures of thoracic spine- |
| S222 | Fracture of sternum- |
| S223 | Fracture of rib- |
| S224 | Multiple fractures of ribs- |
| S225 | Flail chest- |
| S228 | Fracture of other parts of bony thorax- |
| S229 | Fracture of bony thorax, part unspecified- |
| S320 | Fracture of lumbar vertebra- |
| S321 | Fracture of sacrum- |
| S322 | Fracture of coccyx- |
| S323 | Fracture of ilium- |
| S324 | Fracture of acetabulum- |
| S325 | Fracture of pubis- |
| S326 | S32-Fracture of lumbar spine and pelvis |
| S327 | Multiple fractures of lumbar spine and pelvis- |
| S328 | Fracture of other and unspecified parts of lumbar spine and pelvis- |
| S329 | S32-Fracture of lumbar spine and pelvis |
| S420 | Fracture of clavicle- |
| S421 | Fracture of scapula- |
| S422 | Fracture of upper end of humerus- |
| S423 | Fracture of shaft of humerus- |
| S424 | Fracture of lower end of humerus- |
| S427 | Multiple fractures of clavicle, scapula and humerus- |
| S428 | Fracture of other parts of shoulder and upper arm- |
| S429 | Fracture of shoulder girdle, part unspecified- |
| S490 | S49-Other and unspecified injuries of shoulder and upper arm |
| S491 | S49-Other and unspecified injuries of shoulder and upper arm |
| S520 | Fracture of upper end of ulna- |
| S521 | Fracture of upper end of radius- |
| S522 | Fracture of shaft of ulna- |
| S523 | Fracture of shaft of radius- |
| S524 | Fracture of shafts of both ulna and radius- |
| S525 | Fracture of lower end of radius- |
| S526 | Fracture of lower end of both ulna and radius- |
| S527 | Multiple fractures of forearm- |
| S528 | Fracture of other parts of forearm- |
| S529 | Fracture of forearm, part unspecified- |
| S590 | S59-Other and unspecified injuries of forearm |
| S591 | S59-Other and unspecified injuries of forearm |
| S592 | S59-Other and unspecified injuries of forearm |
| S628 | S62.8 Fracture of other and unspecified parts of wrist and hand |
| S629 | S62-Fracture at wrist and hand level |
| S720 | Fracture of neck of femur- |
| S721 | Pertrochanteric fracture- |
| S722 | Subtrochanteric fracture- |
| S723 | Fracture of shaft of femur- |
| S724 | Fracture of lower end of femur- |
| S725 | S72-Fracture of femur |
| S726 | S72-Fracture of femur |
| S727 | Multiple fractures of femur- |
| S728 | Fractures of other parts of femur- |
| S729 | Fracture of femur, part unspecified- |
| S790 | S79-Other and unspecified injuries of hip and thigh |
| S791 | S79-Other and unspecified injuries of hip and thigh |
| S820 | Fracture of patella- |
| S821 | Fracture of upper end of tibia- |
| S822 | Fracture of shaft of tibia- |
| S823 | Fracture of lower end of tibia- |
| S824 | Fracture of fibula alone- |
| S825 | Fracture of medial malleolus- |
| S826 | Fracture of lateral malleolus- |
| S827 | Multiple fractures of lower leg- |
| S828 | Fractures of other parts of lower leg- |
| S829 | Fracture of lower leg, part unspecified- |
| S890 | S89-Other and unspecified injuries of lower l |
| S891 | S89-Other and unspecified injuries of lower l |
| S892 | S89-Other and unspecified injuries of lower l |
| S893 | S89-Other and unspecified injuries of lower l |
| T02 | T02 Fractures involving multiple body regions |
| T020 | T02.0 Fractures involving head with neck |
| T021 | Fractures involving thorax with lower back and pelvis-closed |
| T022 | T02.2 Fractures involving multiple regions of one upper limb |
| T023 | Fractures involving multiple regions of one lower limb-closed |
| T024 | Fractures involving multiple regions of both upper limbs-closed |
| T025 | Fractures involving multiple regions of both lower limbs-closed |
| T026 | T02.6 Fract invol multiple regions of up limb(s) with low limb(s) |
| T027 | T02.7 Fract invol thorax with lower back and pelvis with limb(s) |
| T028 | Fractures involving other combinations of body regions- |
| T029 | T02.9 Multiple fractures, unspecified |
| T08 | T08 Fracture of spine level unspecified |
| T10 | T10 Fracture of upper limb level unspecified |
| T12 | T12 Fracture of lower limb level unspecified |
| T142 | Fracture of unspecified body region- |
| T148 | Other injuries of unspecified body region- |
| T840 | Mechanical complication of internal joint prosthesis- |
| Z87310 | Z87-Personal history of other diseases and conditions |
| Z87311 | Z87-Personal history of other diseases and conditions |

ICD10=International Classification of Diseases 10^th^ edition, MOF=major osteoporotic fracture.

## Table S2: The association between organisational factors and anti-osteoporosis medication (AOM) recorded during hospital stay, accounting for patient case-mix

|  | Organisational factor (range or categories in brackets) | Organisational factor adjusted for case mix and other factors, OR (95%CI) p | | | Organisational factor with case-mix OR (95% CI) | Organisational factor without case-mix OR (95% CI) | Data source |
| --- | --- | --- | --- | --- | --- | --- | --- |
|  | **Factors with p-value<0.1** |  |  |  |  |  |  |
|  |  |  |  |  |  |  |  |
| Pre-op | Proportion of patients admitted to ortho. ward within 4 hours of presentation to ED (Greater than 52% vs 52% or below) | 0.92 | (0.88, 0.96) | <0.001 | 0.95 (0.92, 0.99) p=0.02 | 0.96 (0.93, 1) p=0.05 | Benchmark |
|  | Proportion of patients assessed with an AMT before their operation (100% vs less than 100%) | 1.07 | (1.03, 1.12) | 0.001 | 1.09 (1.05, 1.13) p<0.001 | 1.09 (1.05, 1.14) p<0.001 | BP |
| Peri-op | Orthopaedic NHFD lead has role reflected in their job plan (Yes vs no/unknown) | 1.06 | (1.02, 1.10) | 0.001 | 1.05 (1.02, 1.09) p=0.005 | 1.05 (1.01, 1.08) p=0.01 | NHFD FA |
|  | Protocol in place to prioritise hip fractures for the start of trauma lists (Yes vs no/not stated) | 1.07 | (1.04, 1.11) | <0.001 | 1.07 (1.03, 1.1) p<0.001 | 1.06 (1.03, 1.1) p<0.001 | NHFD FA |
| Post-op | Proportion of patients assessed by a physiotherapist (100% vs less than 100%) | 1.03 | (1.00, 1.06) | 0.048 | 1.05 (1.02, 1.07) p=0.001 | 1.06 (1.03, 1.08) p<0.001 | BP |
|  | Proportion of patients not delirious post-op (Greater than 75% vs 75% or below) | 1.06 | (1.01, 1.10) | 0.009 | 1.06 (1.02, 1.11) p=0.002 | 1.06 (1.02, 1.1) p=0.003 | KPI |
|  | Proportion of patients receiving a bone health assessment during admission (100% vs less than 100%) | 1.09 | (1.06, 1.13) | <0.001 | 1.11 (1.07, 1.15) p<0.001 | 1.1 (1.06, 1.13) p<0.001 | BP |
|  | Model of care (Orthogeriatric models of care vs Traditional orthopaedic care) | 4.65 | (2.25, 9.59) | <0.001 | 4.36 (2.12, 8.99) p<0.001 | 4 (2.01, 7.97) p<0.001 | NHFD FA |
| Governance | Hip fracture service has undertaken QI work in the last year (Yes vs no/not stated) | 0.87 | (0.83, 0.91) | <0.001 | 0.88 (0.84, 0.91) p<0.001 | 0.89 (0.86, 0.93) p<0.001 | NHFD FA |
|  | T&O manager attends clinical governance meeting (Yes vs no/not stated) | 0.90 | (0.87, 0.93) | <0.001 | 0.93 (0.91, 0.96) p<0.001 | 0.94 (0.92, 0.97) p<0.001 | NHFD FA |
|  | Pharmacist attends clinical governance meeting (Yes vs no/not stated) | 0.90 | (0.85, 0.95) | <0.001 | 0.9 (0.86, 0.95) p<0.001 | 0.9 (0.86, 0.94) p<0.001 | NHFD FA |
|  | Physiotherapist attends clinical governance meeting (Yes vs no/not stated) | 0.96 | (0.92, 1.00) | 0.034 | 0.95 (0.92, 0.98) p=0.001 | 0.96 (0.93, 0.98) p=0.003 | NHFD FA |
|  | Clinical governance meetings occur monthly (Yes vs no/not stated) | 1.06 | (1.02, 1.11) | 0.003 | 1.01 (0.97, 1.05) p=0.54 | 1.02 (0.98, 1.06) p=0.43 | NHFD FA |
|  | Consultant anaesthetist attends clinical governance meeting (Yes vs no/not stated) | 1.11 | (1.07, 1.16) | <0.001 | 1.04 (1, 1.07) p=0.03 | 1.03 (1, 1.06) p=0.07 | NHFD FA |
|  |  |  |  |  |  |  |  |
|  | **Factors with p-value>=0.1** |  |  |  |  |  |  |
| Pre-op | Protocol in place for pre-op. energy supplement juice drinks (Yes vs no/not stated) | 1.23 | (0.94, 1.61) | 0.139 | 1.14 (0.86, 1.5) p=0.36 | 1.13 (0.87, 1.47) p=0.36 | NHFD FA |
| Post-op | Proportion of patients returning to original residence (Greater than 72% vs 72% or below) | 1.03 | (0.98, 1.08) | 0.260 | 1.07 (1.02, 1.12) p=0.01 | 1.06 (1.01, 1.11) p=0.02 | KPI |
|  | Hospital reports patients followed up at 120 days (At least some follow-up vs no follow-up or missing) | 1.04 | (0.99, 1.09) | 0.112 | 1.03 (0.99, 1.08) p=0.18 | 1.04 (0.99, 1.08) p=0.13 | Benchmark |
| Governance | Community rehab. team attends clinical governance meeting (Yes vs no/not stated) | 1.00 | (0.94, 1.07) | 0.990 | 1.06 (0.99, 1.13) p=0.07 | 1.07 (1, 1.13) p=0.04 | NHFD FA |
| Workload | Number of hip fracture admissions (recorded in NHFD) per month (Greater than 27 vs 27 or below) | 0.98 | (0.94, 1.01) | 0.138 | 0.97 (0.94, 1) p=0.1 | 0.98 (0.94, 1.01) p=0.12 | NHFD Charts |
|  | Hours of orthogeriatric consultant direct clinical care per week (Greater than 12 vs 12 or below) | 0.98 | (0.93, 1.04) | 0.521 | 1 (0.95, 1.05) p=0.93 | 1 (0.95, 1.05) p=0.97 | NHFD FA |

N=178,470, OR>1 indicates more likely have anti-osteoporosis medication prescribed.

Organisational factors adjusted for case-mix (age group, sex, ASA classification, hip fracture type, pre-fracture residence, and pre-fracture mobility) and mutually adjusted for all backward selected factors shown in table. Red text indicates reversal of effect direction in simpler models.

AMT=abbreviated mental test, ASA=American Society of Anesthesiologists, BP=Best Practice, CI=confidence interval, ED=emergency department, FA=facilities audit, KPI=key performance indicators, NHFD=National Hip Fracture Database, op=operative, OR=odds ratio, ortho.=orthopaedic, QI=quality improvement, rehab.=rehabilitation, T&O=trauma & orthopaedics.

## Table S3: The association between organisational factors and anti-osteoporosis medication (AOM) categories recorded during hospital stay, N= 178,470

|  | Organisational factor |  | Assessed - no AOM needed/appropriate | No assessment or action taken | On no treatment - pending DXA scan or osteoporosis clinic assessment | Continued from pre-admission - oral medication | Continued from pre-admission - injectable medication | Started on this admission - oral medication | Started on this admission - injectable medication |
| --- | --- | --- | --- | --- | --- | --- | --- | --- | --- |
|  | **Factors with p-value<0.1** | **Category** |  |  |  |  |  |  |  |
| Pre-op | Proportion of patients admitted to ortho. ward within 4 hours of presentation to ED | 52% or below | 20.4 | 3.1 | 18.2 | 6 | 0.9 | 41.8 | 9.6 |
|  |  | Greater than 52% | 25 | 3 | 19.1 | 6.4 | 0.9 | 39.2 | 6.5 |
|  | Proportion of patients assessed with an AMT before their operation | Less than 100% | 21.6 | 3.3 | 18.6 | 6.1 | 0.9 | 40.5 | 9.1 |
|  |  | 100% | 20.9 | 1.6 | 16.9 | 6.4 | 0.7 | 46.6 | 6.8 |
| Peri-op | Orthopaedic NHFD lead has role reflected in their job plan | No/unknown | 20.3 | 3.5 | 18.7 | 6.4 | 1 | 40.4 | 9.7 |
|  |  | Yes | 22.2 | 2.8 | 18.2 | 6 | 0.8 | 41.6 | 8.3 |
|  | Protocol in place to prioritise hip fractures for the start of trauma lists | No/not stated | 22 | 3.7 | 19.2 | 5.8 | 1 | 38.1 | 10.2 |
|  |  | Yes | 21.3 | 2.8 | 18 | 6.2 | 0.8 | 42.7 | 8.1 |
| Post-op | Proportion of patients assessed by a physiotherapist | Less than 100% | 21.7 | 3.2 | 18.5 | 5.8 | 0.8 | 40.9 | 9.1 |
|  |  | 100% | 20.9 | 2.8 | 18.2 | 7 | 1 | 41.9 | 8.2 |
|  | Proportion of patients not delirious post-op | 75% or below | 21.5 | 3.5 | 18.3 | 6.1 | 0.9 | 40.7 | 9.1 |
|  |  | Greater than 75% | 21.6 | 1.7 | 18.7 | 6.3 | 0.8 | 42.9 | 8 |
|  | Proportion of patients receiving a bone health assessment during admission | Less than 100% | 21.6 | 3.8 | 18.7 | 6.1 | 0.9 | 40.4 | 8.5 |
|  |  | 100% | 20.9 | 0.3 | 17 | 6 | 0.9 | 44.5 | 10.3 |
|  | Model of care | Traditional orthopaedic care | 19.8 | 18.1 | 36.7 | 6.9 | 0.6 | 16.6 | 1.3 |
|  |  | Orthogeriatric models of care | 21.6 | 2.6 | 17.8 | 6.1 | 0.9 | 42 | 9.1 |
| Governance | Hip fracture service has undertaken QI work in the last year | No/not stated | 20.7 | 3.7 | 17.6 | 5.9 | 0.7 | 46 | 5.5 |
|  |  | Yes | 21.7 | 2.9 | 18.6 | 6.2 | 0.9 | 39.8 | 9.8 |
|  | T&O manager attends clinical governance meeting | No/not stated | 19 | 4.6 | 19.5 | 6.5 | 0.9 | 40.4 | 8.9 |
|  |  | Yes | 22.6 | 2.4 | 17.9 | 5.9 | 0.9 | 41.5 | 8.8 |
|  | Pharmacist attends clinical governance meeting | No/not stated | 21 | 3.4 | 18.6 | 6.2 | 0.8 | 41.9 | 8.2 |
|  |  | Yes | 25.8 | 0.9 | 17.1 | 5.6 | 1.3 | 35.4 | 14 |
|  | Physiotherapist attends clinical governance meeting | No/not stated | 19.3 | 5.1 | 17.7 | 6.9 | 0.8 | 43 | 7.2 |
|  |  | Yes | 22.3 | 2.4 | 18.6 | 5.8 | 0.9 | 40.5 | 9.4 |
|  | Clinical governance meetings occur monthly | No/not stated | 24.8 | 3.9 | 20.4 | 6.2 | 0.7 | 38.1 | 5.9 |
|  |  | Yes | 19.9 | 2.7 | 17.4 | 6.1 | 1 | 42.7 | 10.3 |
|  | Consultant anaesthetist attends clinical governance meeting | No/not stated | 20.3 | 4.2 | 17.5 | 6.1 | 0.9 | 42.6 | 8.5 |
|  |  | Yes | 22.1 | 2.6 | 18.9 | 6.1 | 0.9 | 40.5 | 9 |

## Table S4: The association between organisational factors and refracture in the year post hip fracture, accounting for patient case-mix

|  | Organisational factor (range or categories in brackets) | Organisational factor adjusted for case mix and other factors, OR (95%CI) p | | | Organisational factor with case-mix, OR (95% CI) | Organisational factor without case-mix, OR (95% CI) | Data source |
| --- | --- | --- | --- | --- | --- | --- | --- |
|  | **Factors with p-value<0.1** |  |  |  |  |  |  |
| Pre-op | Orthogeriatric NHFD lead role reflected in job plan (Yes vs no/unknown) | 0.94 | (0.88, 0.99) | 0.030 | 0.94 (0.88, 1) p=0.04 | 0.93 (0.87, 0.99) p=0.03 | NHFD FA |
| Peri-op | Proportion of patients assessed by an orthogeriatrician within 72 hours of admission (100% vs less than 100%) | 0.82 | (0.69, 0.98) | 0.030 | 0.84 (0.71, 1) p=0.06 | 0.84 (0.71, 1) p=0.06 | BP |
| Post-op | Patients in hospital receive physiotherapy on Saturday and/or Sunday (Yes vs no weekend physio) | 0.92 | (0.86, 0.97) | 0.004 | 0.94 (0.88, 1) p=0.05 | 0.94 (0.88, 1) p=0.06 | NHFD FA |
|  | Report of patients followed up at 120 days (At least sone follow-up vs no follow-up or missing) | 1.10 | (1.03, 1.17) | 0.004 | 1.08 (1.01, 1.15) p=0.02 | 1.08 (1.01, 1.15) p=0.02 | Benchmark |
|  | Data submitted for average physiotherapy time received in first week post-op. (Any minutes vs missing) | 1.10 | (1.03, 1.18) | 0.004 | 1.08 (1.01, 1.16) p=0.03 | 1.08 (1.01, 1.15) p=0.03 | PHFSA |
|  | No. of days between discharge and start of community therapy reported (Between 15 and 86 days vs less than 15 or missing) | 1.15 | (1.03, 1.29) | 0.013 | 1.16 (1.04, 1.28) p=0.01 | 1.15 (1.04, 1.28) p=0.01 | PHFSA |
| Governance | OT attends clinical governance meeting (Yes vs no/not stated) | 0.93 | (0.88, 0.98) | 0.007 | 0.97 (0.92, 1.01) p=0.15 | 0.97 (0.92, 1.01) p=0.13 | NHFD FA |
|  | Consultant orthogeriatrician attends clinical governance meeting (Yes vs no/not stated) | 1.07 | (1.01, 1.14) | 0.022 | 1.02 (0.97, 1.08) p=0.36 | 1.02 (0.97, 1.08) p=0.42 | NHFD FA |
|  |  |  |  |  |  |  |  |
|  | **Factors with p-value>=0.1** |  |  |  |  |  |  |
| Peri-op | Proportion of surgery which is NICE compliant (Between 66 and 75% vs 66% or less) | 0.96 | (0.89, 1.02) | 0.185 | 0.98 (0.92, 1.03) p=0.41 | 0.98 (0.93, 1.04) p=0.47 | KPI |
|  | Proportion of surgery which is NICE compliant (More than 75% vs 66% or less) | 0.97 | (0.91, 1.03) | 0.279 | 1 (0.94, 1.05) p=0.86 | 0.99 (0.94, 1.05) p=0.75 | KPI |
|  | Proportion of eligible patients receiving a total hip replacement (Greater than 30 vs 30 or below) | 1.01 | (0.96, 1.06) | 0.702 | 1.02 (0.98, 1.07) p=0.32 | 1.02 (0.97, 1.07) p=0.42 | NHFD Charts |
|  | Proportion of times surgery supervised by consultant surgeon and anaesthetist (Greater than 40 vs 40 or below) | 1.04 | (0.98, 1.10) | 0.195 | 1.02 (0.97, 1.09) p=0.43 | 1.02 (0.96, 1.08) p=0.53 | Benchmark |
| Post-op | Average duration of community therapy in first week (Any minutes vs missing) | 0.99 | (0.92, 1.06) | 0.788 | 1.06 (0.99, 1.13) p=0.1 | 1.05 (0.99, 1.13) p=0.12 | PHFSA |
|  | Proportion of patients returning to original residence (Greater than 76 vs 76 or below) | 1.04 | (0.99, 1.10) | 0.130 | 1.04 (0.98, 1.1) p=0.24 | 1.04 (0.98, 1.1) p=0.22 | KPI |
| Workload | Number of hip fracture admissions (recorded in NHFD) per month (Greater than 27 vs 27 or below) | 1.00 | (0.95, 1.05) | 0.906 | 1 (0.95, 1.05) p=0.9 | 1 (0.95, 1.05) p=0.97 | NHFD Charts |
|  | Proportion of hip fractures occurring in inpatients (Between 3.7 and 5.1% vs 3.7% or less) | 1.03 | (0.99, 1.09) | 0.165 | 1.02 (0.98, 1.07) p=0.34 | 1.02 (0.98, 1.07) p=0.34 | NHFD Charts |
|  | Proportion of hip fractures occurring in inpatients (More than 5.1% vs 3.7% or less) | 1.04 | (0.99, 1.10) | 0.118 | 1.02 (0.97, 1.07) p=0.43 | 1.02 (0.97, 1.07) p=0.47 | NHFD Charts |

N= 178,757, OR>1 indicates increased risk of refracture.

Organisational factors adjusted for case-mix (age group, sex, ASA classification, hip fracture type, pre-fracture residence, and pre-fracture mobility) and mutually adjusted for all backward selected factors shown in table. Red text indicates reversal of effect direction in simpler models.

ASA=American Society of Anesthesiologists, BP=Best Practice, CI=confidence interval, FA=facilities audit, KPI=key performance indicators, NHFD=National Hip Fracture Database, NICE=National Institute for Clinical Excellence, op=operative, OR=odds ratio, OT=occupational therapist, PHFSA=physiotherapy hip fracture sprint audit

## Table S5: The association between organisational factors and refracture in the year post hip fracture N= 178,757

|  | Organisational factors |  | % Refracture | |
| --- | --- | --- | --- | --- |
|  |  |  | **No** | **Yes** |
|  | **Factors with p-value<0.1** | **Category** |  |  |
| Pre-op | Orthogeriatric NHFD lead role reflected in job plan | No/unknown | 92.8 | 7.2 |
|  |  | Yes | 93.3 | 6.7 |
| Peri-op | Proportion of patients assessed by an orthogeriatrician within 72 hours of admission | Less than 100% | 93.1 | 6.9 |
|  |  | 100% | 94.2 | 5.8 |
| Post-op | Patients in hospital receive physiotherapy on Saturday and/or Sunday | No weekend physio | 92.9 | 7.1 |
|  |  | Yes | 93.3 | 6.7 |
|  | Report of patients followed up at 120 days | No follow-up or missing | 93.6 | 6.4 |
|  |  | At least some follow-up | 93.1 | 7.0 |
|  | Data submitted for average physiotherapy time received in first week post-op. | Missing | 93.5 | 6.5 |
|  |  | Any minutes | 93.0 | 7.0 |
|  | No. of days between discharge and start of community therapy reported | Less than 15 or missing | 93.2 | 6.8 |
|  |  | Between 15 and 86 days | 92.4 | 7.6 |
| Governance | OT attends clinical governance meeting | No/not stated | 93.1 | 6.9 |
|  |  | Yes | 93.2 | 6.8 |
|  | Consultant orthogeriatrician attends clinical governance meeting | No/not stated | 93.3 | 6.7 |
|  |  | Yes | 93.1 | 6.9 |
